# Transient Photoactivation of Rac1 Induces Persistent Structural LTP Independent of CaMKII in Hippocampal Dendritic Spines

**DOI:** 10.1523/ENEURO.0263-25.2025

**Published:** 2025-11-26

**Authors:** Takeo Saneyoshi, Chisato Suematsu, Yasunori Hayashi

**Affiliations:** Department of Pharmacology, Kyoto University Graduate School of Medicine, Kyoto 606-8501, Japan

**Keywords:** actin cytoskeleton, CaMKII, dendritic spine, hippocampus, LTP, photoactivation, Rac1, structural plasticity, two-photon microscopy

## Abstract

Structural changes in dendritic spines underlie long-term potentiation (LTP). While CaMKII has been considered as the primary driver of these changes, we show that transient, localized activation of Rac1 alone is sufficient to induce structural LTP in hippocampal slices prepared from rat pups of either sex. Using photoactivatable Rac1 (PA-Rac1), we demonstrated that Rac1 activation triggers spine enlargement and actin polymerization. This PA-Rac1-induced plasticity was blocked by Rac1 and Pak1 inhibitors but not by a CaMKII inhibitor. Our results identify Rac1 as an upstream of persistent signaling that stabilizes actin-based spine structural changes critical for synaptic memory encoding.

## Significance Statement

The molecular mechanisms that trigger persistent structural long-term potentiation (sLTP) at synapses remain incompletely understood. This study demonstrated that localized activation of Rac1, a small GTPase regulating actin dynamics, is sufficient to induce and maintain sLTP in hippocampal neurons independently of CaMKII. Using two-photon photoactivation and fluorescence lifetime imaging microscopy (FLIM), we show that Rac1 induces persistent spine growth and actin polymerization. These findings identify Rac1 as a self-sustaining signaling module in synaptic plasticity and provide mechanistic insight into the biochemical encoding of long-lasting synaptic changes that underlie memory.

## Introduction

Long-term potentiation (LTP) is a fundamental synaptic mechanism underlying learning and memory. While CaMKII has been widely studied as a key kinase capable of the sustained activity required for LTP maintenance (Yasuda et al., 2022), how its signaling is translated into enduring structural changes in dendritic spines remains incompletely understood.

A CaMKII-Tiam1 complex, termed reciprocal activation of kinase-effector complex (RAKEC), was previously shown to generate persistent activation of Rac1, a Rho family small GTPase, and promote spine enlargement during structural LTP (sLTP; Saneyoshi et al., 2019; Saneyoshi, 2021). Through activation of a kinase cascade eventually leading to the inactivation of the actin severing protein cofilin1, Rac1 regulates dendritic spine morphology (Saneyoshi and Hayashi, 2012), synaptic plasticity (Haditsch et al., 2009), motor learning (Hayashi-Takagi et al., 2015), and forgetting (Liu et al., 2016). In non-neuronal cells, Rac1 activity can be stabilized by positive feedback through the coronin 1A-based multiprotein complex (Castro-Castro et al., 2011), raising the possibility that Rac1 may serve as a persistent signaling module downstream of CaMKII.

To directly test this, we used photoactivatable Rac1 (PA-Rac1; Wu et al., 2009) in hippocampal slice cultures to precisely manipulate Rac1 activity in single dendritic spines and assess whether Rac1 alone can induce and maintain sLTP. We found that a transient activation of Rac1 can induce persistent increases in F-actin and enlargement of dendritic spines. This indicates that the mechanism that maintains the enlarged dendritic structure of dendritic spines lies downstream to Rac signaling, thereby opening new future direction of LTP research.

## Materials and Methods

### Plasmids

Photoactivatable Rac1 (PA-Rac1; Addgene plasmid #22035) was a gift from Dr. Klaus Hahn (Wu et al., 2009) and was subcloned into the pCAGGS expression vector (Niwa et al., 1991). Lifeact was obtained from Dr. Wedlich-Söldner (Riedl et al., 2008). Constructs encoding monomeric GFP, photoactivatable GFP (PAGFP), DsRed2, human Rac1, and β-actin have been described previously (Saneyoshi et al., 2008; Bosch et al., 2014).

### Reagents

KN-93, EHT1864, IPA3, and MNI-caged ʟ-glutamate were purchased from Tocris Bioscience. Picrotoxin was obtained from Nacalai Tesque, and tetrodotoxin (TTX) was from Latoxan. Latrunculin and Jasplakinolide were from FUJI-FILM.

#### Organotypic slice culture and transfection

Organotypic hippocampal slices cultures were prepared from postnatal day 6–9 Sprague Dawley rat pups of both sexes (Stoppini et al., 1991). At 7–8 d in vitro, cultured slices were biolistically transfected with plasmid DNA using a Gene-Gun (Bio-Rad) at 180 psi, as described previously (Saneyoshi et al., 2019). For each transfection, 10 µg of plasmid DNA was coated onto 12.5 mg of 1.6 µm gold particles.

#### Two-photon imaging and structural LTP induction

Imaging was performed using a two-photon laser scanning microscope (FV1000-MPE, Olympus) equipped with Ti-sapphire pulsed lasers (Spectra-Physics). Slices were continuously perfused at room temperature (25–27°C) in artificial cerebrospinal fluid (ACSF) containing the following (in mM): 119 NaCl, 2.5 KCl, 4 CaCl_2_, 26.2 NaHCO_3_, 1 NaH_2_PO_4_, and 11 glucose, equilibrated with 95% O_2_/5% CO_2_. For glutamate uncaging experiments, ACSF was supplemented with 1 µM TTX, 50 µM picrotoxin, and 2.5 mM MNI-ʟ-glutamate. For PA-Rac1 experiments, ACSF contained 1 mM MgCl_2_, 2.5 mM CaCl_2_, and 1 µM TTX.

Pharmacological inhibitors were bath-applied at least 30 min before stimulation. Structural LTP was induced by two-photon uncaging of MNI-glutamate at 720 nm (30 pulses, 2 ms duration, 1 Hz) with 5 mW power at the specimen. PA-Rac1 was photoactivated using a 720 nm laser (20 pulses, 0.5 ms duration, 1 Hz). Imaging was performed at 910 nm for uncaging experiments and 1,030 nm for PA-Rac1 imaging to avoid nonspecific Rac1 activation. Spine volume was quantified using the total integrated fluorescent intensity of the GFP or DsRed2 in *z*-stacked images of spines with background subtraction by FIJI (Schindelin et al., 2012).

#### Fluorescence lifetime imaging microscopy

Fluorescence lifetime was measured using time-correlated photon-counting (SPC-150 module, Becker-Hickl; H7422P-40 detector, Hamamatsu Photonics), as previously described (Saneyoshi et al., 2019). Data were analyzed using a custom written macro in Igor-Pro (WaveMetrics). Lifetime values were averaged within spine heads and normalized as changes from baseline. For actin polymerization, FRET-FLIM imaging was performed using mVenus- or mGFP-Actin and Lifeact-mRFP.

### Statistical analysis

All data are presented as mean ± SEM. For comparison between two groups, unpaired two-tailed *t* tests were used. For multiple groups comparison, one-way ANOVA with Tukey's post hoc test was applied. A significance threshold was set at *p* < 0.05. Sample size (*n*) refers to individual neurons as specified in figure legends.

## Results

**Figure 1.**
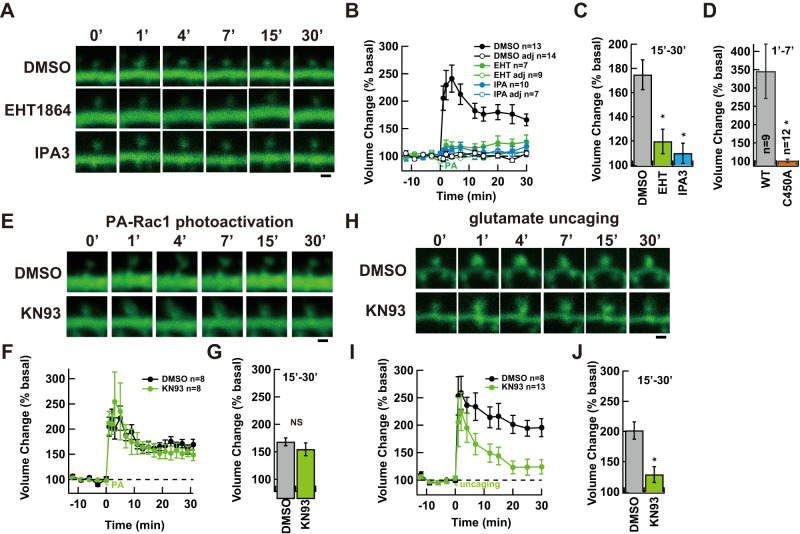
Rac1-dependent induction of structural LTP. ***A***, Representative images PA-Rac1-induced structural LTP in organotypic hippocampal slice culture expressing GFP and PA-Rac1, following photoactivation under control (DMSO, 0.1%), Rac inhibitor (EHT1864, 50 µM), or Pak inhibitor (IPA3, 30 µM) conditions. ***B***, Time course of spine volume changes during PA-Rac1 activation (DMSO, black; EHT1864, green; IPA3, blue). ***C***, Quantification of spine volume in the persistent phase (15–30 min) under each condition (mean ± SEM; *n* = 13 neurons for DMSO, *n* = 7 neurons for EHT1864, *n* = 10 for IPA3). ***D***, Quantification of spine volume in initial phase (1–7 min) using PA-Rac1 WT or LOV2 dark mutant, C450A (mean ± SEM; *n* = 9 neurons for WT, *n* = 12 neurons for C450A). ***E***, Representative images showing PA-Rac1-induced sLTP in the presence of DMSO or the CaMKII inhibitor KN93 (10 µM). ***F***, Time course of spine volume changes (DMSO, black; KN93, green). ***G***, Comparison of spine volume during the persistent phase (15–31 min) between DMSO and KN93 (mean ± SEM; *n* = 8 neurons per group). ***H***, Representative images showing glutamate uncaging-induced sLTP in the presence of DMSO (0.1%) or the CaMKII inhibitor KN93 (10 µM). ***I***, Time course of spine volume changes (DMSO, black; KN93, green). ***J***, Comparison of spine volume during the persistent phase (15–30 min) between DMSO and KN93 (mean ± SEM; *n* = 8 neurons for DMSO; *n* = 13 neurons for KN93). Statistical analysis: two-way ANOVA with post hoc Tukey’s test for ***C***; unpaired two-tailed *t* tests for ***D***, ***G***, and ***J***; *p < 0.05; NS, not significant; Scale bars, 1 µm.

To directly test whether Rac1 activation is sufficient for sLTP, we used a photoactivatable Rac1 (PA-Rac1; Wu et al., 2009) in CA1 pyramidal neurons of organotypic hippocampal slice cultures. This system enabled precise spatial and temporal control of Rac1 activation through light-induced conformational change of a phototropin protein, LOV2, from *Avena sativa*. We expressed mCherry-PA-Rac1 with GFP as a volume filler using a biolistic method and imaged the expressing neurons using two-photon laser scanning microscopy (Saneyoshi et al., 2019). Our initial attempt of imaging using 910 nm light induced ectopic lamellipodia during repeated imaging, likely due to nonspecific PA-Rac1 activation by the imaging laser. We therefore performed imaging at 1,030 nm to prevent such activation (data not shown). Spine-specific Rac1 activation was achieved by two-photon activation at 720 nm (0.5 ms pulses 20 repetitions at 1 Hz), which resulted in rapid and sustained spine enlargement persisting up to 30 min (Fig. 1*A–C*). The volume of unstimulated adjacent spines nearby the stimulated spine did not change, indicating specificity of photoactivation of PA-Rac1 (Fig. 1*B*). A mutant mimicking dark state (LOV2 C450A) failed to induce enlargement, confirming the specificity of PA-Rac1-mediated effects (Fig. 1*D*).

Next, we tested whether Rac1-induced structural enlargement depends on the canonical Rac signaling pathway. Inhibition of Rac1 with EHT1864 (Shutes et al., 2007), or its downstream effector Pak1 with IPA3 (Deacon et al., 2008) blocked both the induction and maintenance phases of spine enlargement following PA-Rac1 activation (Fig. 1*A–C*). These results confirm that the Rac1-Pak1 signaling pathway is both necessary and sufficient for sustained structural spine plasticity, consistent with previous chemical-genetic studies of Pak1 and its effector LIMK1 (Dagliyan et al., 2017; Ripoli et al., 2023). In contrast, inhibition of CaMKII with KN93 (Sumi et al., 1991) has no effect on PA-Rac1-induced sLTP (Fig. 1*E–G*), indicating that once Rac1 is activated, structural LTP can proceed independently of ongoing CaMKII activity. The KN93 inhibited sLTP induced by glutamate uncaging, confirming the effect of the inhibitor (Fig. 1*H–J*).

**Figure 2.**
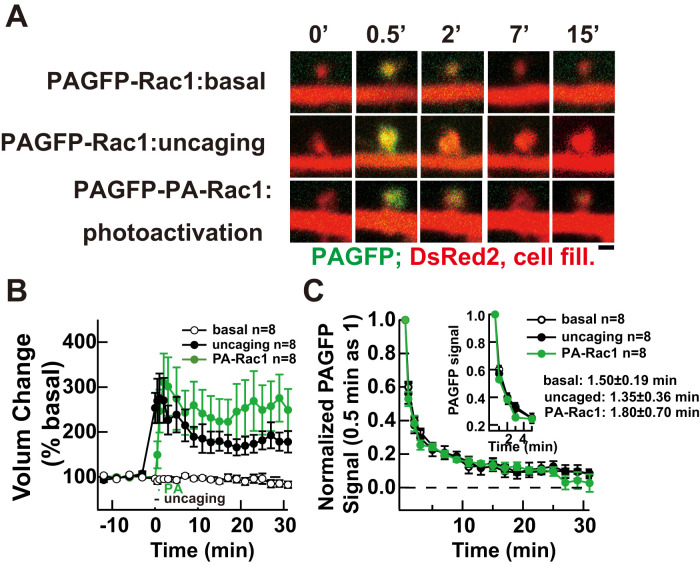
Molecular dynamics of Rac1 and PA-Rac1 in dendritic spines during sLTP. ***A***, Representative merged images showing spine morphology (DsRed2) and PAGFP fluorescence for Rac1 or PA-Rac1 under basal conditions (top), uncaging-induced sLTP (middle), or PA-Rac1 photoactivation (bottom). ***B***, Quantification of normalized spine volume changes across conditions (PA-Rac1, green; basal, white; uncaging-induced sLTP, black; *n* = 8 neurons per group). ***C***, Normalized PAGFP decay curves showing fluorescence intensity divided by spine volume at 0.5 min poststimulation: Rac1 in basal (white, *τ* = 1.50 ± 0.19 min), Rac1 in glutamate uncaging-induced sLTP (black, *τ* = 1.35 ± 0.36 min), photoactivated PA-Rac1 (green, *τ* = 1.80 ± 0.70 min). Scale bars, 1 µm.

Two factors determine the activity profile of PA-Rac1 after photoactivation: (1) intrinsic properties of the LOV2 domain conformational changes and (2) the diffusion of the PA-Rac1 protein out of dendritic spine. The LOV2 domain undergoes conformational changes within seconds of light illumination and returns to the dark state within ∼1 min (Wu et al., 2009; Zoltowski et al., 2009). To assess the contribution of protein diffusion, we analyzed the fluorescence decay of PAGFP-tagged PA-Rac1 and Rac1 within dendritic spines following local photoactivation. All conditions—PAGFP-Rac1 under basal condition, PAGFP-Rac1 in glutamate uncaging stimulated spines, and PAGFP-PA-Rac1 following photoactivation—showed comparable fluorescence decay kinetics (*τ* ≈ 1.35–1.80 min), suggesting similar mobility regardless of activation state (Fig. 2*A–C*). Although not statistically significant, there was a trend toward slower decay for activated PA-Rac1, consistent with previous findings that the active Rac1 mutant shows reduced diffusion in spines (Chazeau et al., 2014). These data suggest that photoactivated Rac1 is quickly lost from dendritic spines due to both intrinsic inactivation of the LOV2 domain and protein diffusion and thus imply that transient Rac1 activity triggers a persistent change specifically in the photoactivated dendritic spine that leads to an enlargement of the structure.

**Figure 3.**
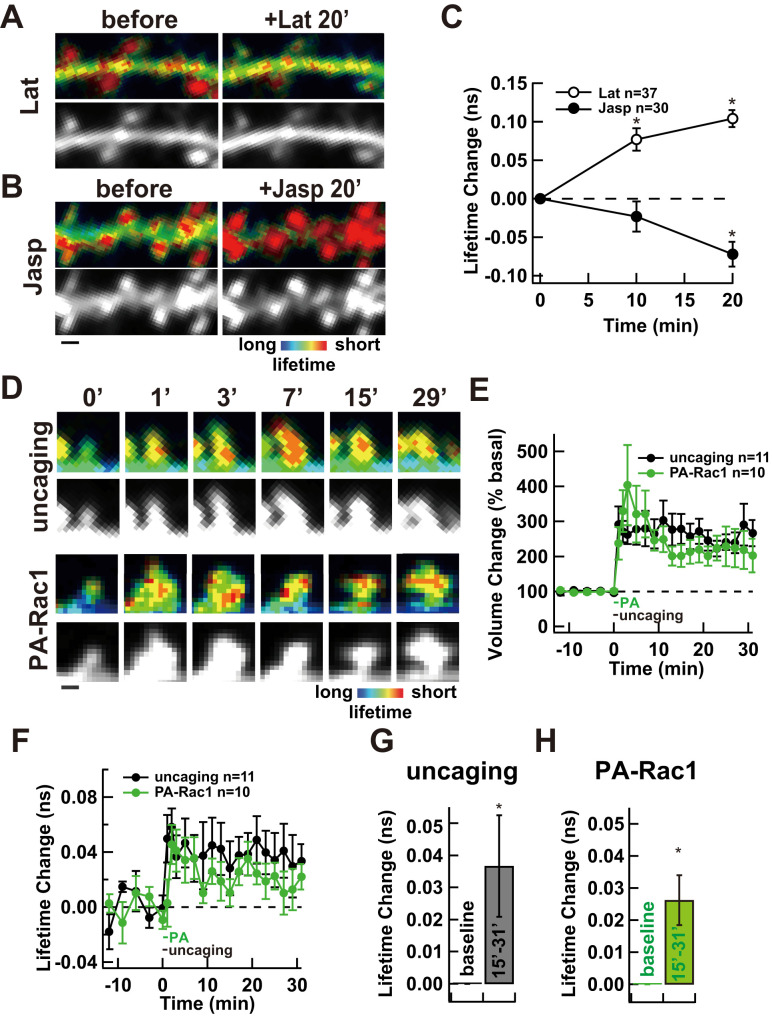
Actin polymerization during uncaging- or PA-Rac1-induced structural LTP. ***A***,*** B***, Representative FRET-FLIM images (top) and spine morphology images (bottom, using the GFP-Actin signal as a proxy) showing actin polymerization before and 20 min after pharmacological treatment with an inhibitor of actin polymerization (latrunculin, 10 µM; ***A***) or an inducer of actin polymerization (jasplakinolide, 1 µM; ***B***). Warmer colors indicate shorter lifetimes, which are indicative of increased actin polymerization. ***C***, Time course of lifetime changes in dendritic spines by pharmacological treatment (open dot: latrunculin, *n* = 37 spines from six neurons; closed dot: jasplakinolide, *n* = 30 spines from five neurons). Statistical analysis: unpaired two-tailed *t* tests; *p < 0.05. Scale bars: 2 µm. ***D***, Representative FRET-FLIM images (top) and spine morphology (bottom, using the GFP-Actin signal as a proxy) showing actin polymerization before and after sLTP induction. Warmer colors indicate shorter lifetimes, indicative of increased actin polymerization. ***E***, Time course spine volume changes during sLTP induced by glutamate uncaging (black, *n* = 11 neurons) or PA-Rac1 activation (green, *n* = 10 neurons). ***F***, Time course of fluorescence lifetime changes during uncaging (black) or PA-Rac1 activation (green). ***G***, ***H***, Comparison of lifetime values at baseline and during the persistent phase (15–31 min) for uncaging-induced sLTP (***G***) or PA-Rac1-induced sLTP (***H***). Statistical analysis: unpaired two-tailed *t* tests; *p < 0.05. Scale bars, 1 µm.

To evaluate actin cytoskeletal remodeling, we developed a novel approach to detect actin polymerization using two-photon fluorescence lifetime imaging microscopy with mGFP- or mVenus-Actin and Lifeact-mRFP, a protein specifically interact with F-actin (Riedl et al., 2008) as FRET pairs. To test if it can indeed detect actin polymerization, we treated neurons with latrunculin, an inhibitor of actin polymerization, or jasplakinolide, an inducer of actin polymerization to observe the actin polymerization and compared the lifetimes changes before and after these treatments. Latrunculin-induced actin depolymerization increased the fluorescence lifetime while jasplakinolide-induced actin polymerization decreased it, indicating that this approach can detect actin polymerization/depolymerization status bidirectionally within dendritic spines (Fig. 3*A–C*).

We then compared actin polymerization/depolymerization status between glutamate uncaging-induced sLTP and PA-Rac1 activation (Fig. 3*D–H*). Both stimuli produced comparable spine enlargement (Fig. 3*E*) and actin polymerization during the maintenance phase of LTP (Fig. 3*F–H*), suggesting that Rac1 activation alone can mimic the structural and actin cytoskeletal remodeling observed with synaptic stimulation.

## Discussion

Our study establishes that localized and transient Rac1 activation is sufficient to induce and maintain structural LTP independently of upstream CaMKII signaling, positioning Rac1 as a trigger of the self-sustaining signal which is required for inducing long-lasting synaptic structural plasticity. While CaMKII is essential for LTP induction, our findings suggest that once Rac1 is activated, it can autonomously maintain spine structure via downstream effectors such as Pak1 and actin remodeling. These findings extend the proposed reciprocal activation of the kinase-effector complex (RAKEC) model, in which CaMKII and Tiam1 form a signaling complex that reciprocally activates both CaMKII and Tiam1-mediated Rac1 activity during LTP. Our results indicate that Rac1, once activated—whether downstream of RAKEC or via PA-Rac1 activation—can maintain plasticity independently. This supports a modular view of synaptic signaling in which RAKEC initiates plasticity through coordinated dual activation, while Rac1 ensures its structural persistence (Hedrick et al., 2016; Saneyoshi et al., 2019).

Recent studies have highlighted that CaMKII contributes to LTP not only through its kinase activity but also via kinase-independent mechanisms (Tullis et al., 2023; Chen et al., 2024; Claiborne et al., 2024). Specifically, CaMKII kinase activity is essential for LTP induction, where autophosphorylation at T286 is the only action to be required (Chen et al., 2024). However, maintenance of LTP appears to depend on kinase-independent roles of CaMKII, such as structural or scaffolding interactions within the postsynaptic density. Although the precise molecular mechanisms remain under debate, this emerging model aligns with our finding that Rac1 activation alone is sufficient to induce sLTP in a CaMKII-independent manner (Fig. 1).

Persistent Rac1 activation during the late phase of LTP may arise through at least two upstream signaling routes: one mediated by the CaMKII-Tiam1 RAKEC mechanism, which sustains Rac1 activity downstream of CaMKII (Saneyoshi et al., 2019), and another involving autocrine BDNF/TrkB signaling (Tep et al., 2012; Harward et al., 2016) and PKC (Tu et al., 2020). Because the RAKEC complex mechanism requires CaMKII kinase activity but not necessarily T286 autophosphorylation, inhibition of CaMKII would be expected to disrupt this pathway—consistent with our observation that direct Rac1 activation can bypass this requirement. Nonetheless, how persistent actin polymerization is maintained during the late phase of LTP remains unresolved. Further studies are needed to clarify how Rac1 signaling integrates with kinase-independent functions of CaMKII to stabilize spine structural plasticity.

A major remaining question is how transient Rac1 activity is converted into persistent signaling that maintains actin remodeling over time. In non-neuronal systems, Rac1 participates in positive feedback loops that maintain its GTP-bound state via the coronin 1A multiprotein complex (Castro-Castro et al., 2011). In neurons, similar mechanisms may exist—such as Rac1-mediated recruitment of Rac-specific GEFs (Penzes et al., 2008), inhibition of Rac-GAPs, or local assembly of a postsynaptic protein complex through mechanisms such as liquid–liquid phase separation (Chen et al., 2017). Such feedback could promote persistent, spatially restricted Rac1 signaling in spines during memory encoding.

The present study did not directly assess whether PA-Rac1-induced sLTP is accompanied by potentiation of AMPAR-mediated synaptic current. However, several lines of evidence suggest that Rac1 activation may be sufficient to promote AMPAR surface expression. For instance, Rac1 activation has been shown to drive AMPAR insertion in developing neurons (Wiens et al., 2005), and phosphorylation of its downstream effector PAK3 enhances AMPAR surface trafficking (Hussain et al., 2015). These findings imply that Rac1-dependent actin polymerization may facilitate the structural remodeling necessary for AMPAR stabilization at the postsynaptic membrane. Thus, combining photoactivatable signaling tools such as PA-Rac1 with electrophysiological or super-resolution imaging approach could provide a powerful strategy to dissect the molecular coupling between actin dynamics and AMPAR recruitment during LTP expression and maintenance.

Our findings also intersect with previous studies implicating Rac1 in both memory formation and forgetting. For example, a prolonged photoactivation of Rac1 (150 ms light pulse at 1 Hz for 1 h) specifically in potentiated synapses after a motor learning task can erase acquired memories (Hayashi-Takagi et al., 2015), suggesting that widespread and prolonged Rac1 activity may promote synapse weakening or elimination. In contrast, our approach—brief, localized photoactivation at single spines—induces long-lasting spine enlargement. This is consisted with in vivo observations in the nucleus accumbens medium spiny neurons, where Rac1 photoactivation using 473 nm light (0.5 ms light pulse at 10 Hz for 10 min) produced a small but significant increase in spine head diameter and mushroom spine density (Wright et al., 2020). These divergences likely reflect differences in the spatial and temporal pattern of Rac1 activation as well as in brain regions. Broad activation may engage signaling crosstalk with RhoA pathways (Wu et al., 2009) or trigger homeostatic plasticity across dendritic segments, shifting the role of Rac1 from potentiation to facilitating synapse pruning.

Finally, our application of a FLIM–FRET biosensor (mGFP-Actin and Lifeact-mRFP; Fig. 3) enabled quantitative, spine-specific monitoring of actin remodeling in live brain tissue. This actin probe system provides a powerful tool to dissect the biochemical dynamics downstream of Rac1 and can be applied to future studies to understand feedback regulation and synaptic remodeling in memory.

## Synthesis

Reviewing Editor: Matthew Grubb, King's College London Decisions are customarily a result of the Reviewing Editor and the peer reviewers coming together and discussing their recommendations until a consensus is reached. When revisions are invited, a fact-based synthesis statement explaining their decision and outlining what is needed to prepare a revision will be listed below. The following reviewer(s) agreed to reveal their identity: NONE.

Both Reviewers are positive about the main findings of the manuscript. However, revisions are required such that the data presented fully support the conclusions of the submission.

In particular, any revised manuscript must:

1) Provide a positive control that CaMKII inhibition was successful (see Reviewer 2's point #2). This should take the form of additional experiments using the exact conditions and batch of antagonist presented in the original submission, unless a very strong, referenced argument can be made that the CaMKII inhibition as used in the original submission was overwhelmingly likely to have worked as intended.

2) If the FRET sensor for polymerised actin is new, provide a positive control/characterisation that it does indeed report polymerised actin (see Reviewer 2's point #4). If it is not new, provide references fully supporting its efficacy in the revised manuscript.

3) Either provide additional analysis demonstrating that PA-Rac1 induces sLTP in a spine-specific manner (i.e. not in neighbouring spines), or remove from the manuscript any claims for spatial specificity (see Reviewer 2's point #3).

In addition, please ensure that all of the other comments from the Reviewers are fully addressed in any revised submission.

Reviewer 1:

This paper makes an important contribution to the mechanistic understanding of the induction of structural LTP. Transient activation of Rac1 was shown to be sufficient to induce sLTP, and surprisingly, this CaMKII activity was not needed. This puts Rac1 activation downstream of CaMKII activation and also indicates that CaMKII is not needed for the maintenance of sLTP.

My only concern is that there are some overinterpretations. In particular, the authors have not shown that Rac1 is required (or sufficient) for LTP maintenance. This can only be shown when Rac1 is manipulated after LTP induction. But this was not done. Basically, the authors cannot exclude the possibility that the transient activation of Rac1 activated a downstream mechanism that maintains LTP. Therefore, the claims of the manuscript need to be adjusted, including the abstract and title of Figure 1.

Additionally, I do not agree with the claim that the authors would have identified Rac1 as a persistent signaling node that stabilises structural changes. In this paper, Rac1 was only transiently activated, and this led to a persistent activation of Rac1 as for glutamate uncaging, but it was not shown here that this stabilises structural changes. Again, to show this, a post-induction manipulation is needed.

Reviewer 2:

In this manuscript, the authors examine the molecular mechanisms that drive the structural plasticity of dendritic spines, a vital component of the cellular mechanisms of learning. Using a photoactivatable Rac1 (PA-Rac1), they demonstrate that activated Rac1 is sufficient to drive long-term dendritic spine enlargement. Using pharmacological approaches, they report that this PA-Rac1 induced spine enlargement is dependent upon Rac1 and its downstream effector, Pak1, but not on CaMKII. Finally, using 2-photon FRET-FLIM with a tagged Lifeact-actin FRET pair, the authors report that photoactivation of Rac1 induces a long-term increase in actin polymerization, similarly to that observed with glutamate uncaging.

Although it has been shown that Rac1 is necessary downstream of CaMKII in driving spine structural plasticity, the authors now report that it is also sufficient. This is perhaps not surprising, as it is downstream of CaMKII; however, it is an interesting conclusion, considering the recent reports on kinase-independent requirements of CaMKII for LTP of AMPA currents, but apparently not for long-term stabilization of mature spine plasticity - it would be worthwhile to address this in the discussion. In addition, there are several important controls and items that should be addressed.

MAJOR

1. The concentrations of the pharmacological agents are missing - they should be reported in the Figure legends.

2. The main conclusion is that Rac1's action is independent of CaMKII; however, this is based on a negative result. Ideally, the authors would show that the CaMKII inhibitor is working, perhaps by inhibiting long-term spine structural plasticity induced by glutamate uncaging.

3. The authors claim that the local activation of PA-Rac1 induces sLTP specifically in the spine targeted for photoactivation; however, quantification of nearby unstimulated spines is not provided. The authors should show nearby unstimulated spines in their images and quantify them in their graphs, or eliminate claims of single spine specificity from the text.

4. The authors present what appears to be a new FRET sensor for polymerized actin. If it is not new, please cite the origin. If it is new, it would be important to perform a pharmacological manipulation on actin to demonstrate that it is reporting polymerized actin. Does the Rac1 inhibitor block the glutamate uncaging-induced polymerization reported by this sensor?

MINOR

5. It has been shown that CaMKII plays a kinase-independent role in long-term synaptic plasticity (Tullis et al., 2023; Chen et al., 2024) and new spine stabilization (Claiborne et al., 2024), but not in long-term stabilization of mature spines (Claiborne et al., 2024). Presumably, Rac1 is downstream of CaMKII kinase activity - the authors should comment on the aspects of CaMKII function that are inhibited by their KN93 treatment (which has been reported to block both kinase activity and inhibit interaction of CaMKII with the NMDAR) and its implications.

6. Figure 2 needs to be described more clearly in the text and labeled better in the Figure. Please indicate what the colors represent - presumably green is PA-GFP and red is the red cell fill? Presumably PA-Rac1 is unlabeled? Perhaps it would help to label the images: PA-GFP basal, PA-GFP uncaging, PA-GFP + PA-Rac 1.

7. The statistics are unclear in Figure 3C. Please present a bar graph in addition to the line graph in order to clarify the statistical comparisons that are being made - is there a comparison between the two conditions or just only with baseline? is missing a legend for the lifetime maps.

8. How is spine volume being quantified? Please describe in the methods. In a related issue, it is unclear which fluorophore is being used to quantify spine volume in figure 3.

9. Miscellaneous small items. Typo on line 99 ("biolithic" should be "biolistic"). Fig 2B the word uncaging on the graph doesn't seem to be associated with anything in particular. Fig. 2C perhaps present a short time period or an inset to better show the Tau. Description of experiments in Wright et al., 2020 on lines 170-171 seems inaccurate - please check.
